# Impact of Navier’s slip and MHD on laminar boundary layer flow with heat transfer for non-Newtonian nanofluid over a porous media

**DOI:** 10.1038/s41598-023-39153-y

**Published:** 2023-08-03

**Authors:** T. Maranna, S. M. Sachhin, U. S. Mahabaleshwar, M. Hatami

**Affiliations:** 1 Department of Studies in Mathematics, Shivagangotri, Davangere University, Davangere, India; 2 Department of Mechanical Engineering, Esfarayen University of Technology, Esfarayen, North Khorasan Iran

**Keywords:** Engineering, Mathematics and computing

## Abstract

The current studies analytically summarize the impact of magnetohydrodynamic and thermal radiation on the non-Newtonian continuous uniform motion of viscid non-compressible nanofluid across a penetrable stretching/shrinking sheet, even though accomplish Navier's first and second order slips along mass transpiration. Blood-bearing silver and copper nanomaterials have distinct flow and heat transfer properties when exposed to heat. Silver (*Ag*) as well as copper (*Cu*) nanoparticles are assumed to be present in blood as the non-Newtonian liquid; this fluid serves as the base. We anticipate that the current study will be useful in fields including food, petrochemical products, and medicines, as well as blood circulation, and highly beneficial for patients who are dealing with blood clotting in the uterus, which may result in infertility or cancer, to evaluate the blood flow in the tube. Employing the similarity conversion technique, the ruling partial differential equations are modified into a couple of non-linear ordinary differential equations. Then the transformed ordinary differential equations are analytically solved with the Laplace transformation and expressed in terms of an incomplete gamma function. The current analytical results are compared to previous studies. It is addressed how several physical features such as magnetic field *M*, Navier’s first and second order slip, permeability, Prandtl number *Pr*, and radiation parameter affect non-dimensional velocity as well as temperature patterns through graphs. The results obtained reveal that there is an enhancement in the rate of heat transfer with the rise in nanoparticle volume fraction and radiation. The temperature distribution is also influenced by the presence of Prandtl numbers, radiation, solid volume fraction, permeability, and slip conditions*.* This shows that the solid volume fraction of nanoparticles can be used to control the behaviour of heat transfer and nanofluid flows.

## Introduction

For the past 20 years, various applications that are presently in use or that are intended have stimulated intensive research towards nanofluids or suspensions with nanoparticles. Although nanoparticles are employed in the flow regime throughout all real-world scenarios, the structure of the flow is dictated by the appropriate thermophysical properties of these nanofluids. Recently, nanofluids have been implemented for industrial and technological purposes. Printing, photovoltaic panels, geothermal power systems, electronic items, nanoscale gadgets for illness, refrigeration of metallic particles, microprocessors, lasers, cardboard boxes, and inkjets are only a few possibilities for nanoparticles. 100-nm-diameter nanocomposites are a mixture of solid and liquid nanostructures. The concept of nanofluids is described by Choi et al.^1^ as being initially used in order to optimize the materials thermal performance. Eastman et al.^2^ developed Copper nanoparticles were immersed in a polyethylene glycol-based nanoliquid that had greater heat stability than almost any other conventional base liquid, particularly ethylene glycol.

Usually, the nanomaterials used within nanoliquids consist of metals (aluminium, copper), oxides (aluminium oxides), nitrides (aluminium nitrate, silicon nitrate), carbides (silicon carbide), or non-metals (CNTs, graphite). Additionally, biological research seems to be employing a variety of fragments, including deoxyribonucleic acid, ribonucleic acid, biomolecules, and fluids articulated in porous materials^3–5^. Researchers focusing on global ecosystems have recently raised thermal conductivity and optimized heat transportation characteristics by mixing two or more nanoparticles into a common base liquid. In works like those by researchers^6–13^, initiatives to address the mobility of nanoparticles under several circumstances as well as their features are shown. The study of connections among electromagnetic forces as well as electrically conducting fluids is described as magnetohydrodynamics. The Swedish researcher Hannes Alves et al.^14^ developed the concept of MHD. Nowadays, magnetic fields are extensively employed in numerous major industries. In refrigeration or heating techniques, the magnetic situation is taken into account to enhance thermal performance. Because of its broad range of implications, the researchers used the magnetic field effect.

The latest study by Mahabaleshwar et al.^15–20^ on the casson hybrid nanofluid, nanofluids, dusty fluids, and carbon nanotubes physical phenomena under the presence of MHD and heat transfer along mass transpiration due to stretching/shrinking surfaces is discussed. Due to its special features, silver has a broad range of biological purposes. Generally, silver-containing products are employed to fight a wide range of bacteria. Additionally, according to verifiable studies, Ag nanoparticles can replace conventional anticancer treatments in a manner that is more ecologically responsible and biodegradable^21^. The most significant biological fluid is blood, which is a fluid made up of many types of cells dispersed in a soluble fluid medium (the plasma). It ought to be emphasized that red blood cells in plasma have a role in rotational motion whenever a relative velocity arises. The body's cells have both an angular orbital momentum and an angular gyration moment. Hence, blood can be conceived as a non-Newtonian fluid with a uniform density. There are a variety of polar fluid hypotheses that can be explored, notably Stokes concept^22^.

Researchers and scientists from all around the universe have been working on various features of nanotechnology over the past few decades. For a bio-nanofluid, Ghassemi et al.^23^ studied a new efficient thermal conductance concept (blood with nanoparticle Al_2_O_3_). A good deal of researchers^24,25^ are investigating theoretical studies that address the non-Newtonian blood circulation in stenotic arteries because capillary membranes are perhaps stretchy, absorptive, and changeable. Moreover, human blood was used as a base fluid to investigate heat convective boundary movements^26,27^. To date, the trustworthy literature regarding recent developments in this fluid flow can be found in Refs.^28–33^.

To the author's best knowledge and inspired by the aforementioned studies and potential implications in the current work, it is important to examine the impacts of magnetohydrodynamics as well as heat radiation on the Navier's first and second order slips along mass transpiration in a non-Newtonian constant laminar stream of viscous incompressible nanofluid across a porous stretching/shrinking surface. By using a similarity conversation, the associated partial differential equations for dominating flow are converted via a system of ordinary differential equations. Analytical approaches are found as a function of nonlinear integrated differential equations.

## Physical problem and governing equation

Considering the flow of incompressible viscous nanoparticles in two dimensions across a porous stretching/shrinking surface. The surface is extended out within the direction of motion as taking on the $x$-axis, with the $y$-axis perpendicular to the slit. A uniform magnetic field $B_{0}$ is applied to the fluid flow. When two equal-strength forces are imposed towards the $x$-axis to restrain the liquid movement in the region $y > 0$. Suppose the sheet is submerged in a fluid with a consistent ambient temperature $T_{\infty }$ as well as a surface temperature $T_{0}$.

Considering that the fluid is inviscid, the principle of mass conservation is simplified^34^ as follows:

1 $$\nabla .\,\overrightarrow {q\,} \, = \,0,$$

together with the principle of conservation of linear momentum defined as^34,35^ following

2 $$\left( {\overrightarrow {{q_{t} }} + \left( {\overrightarrow {q} .\,\nabla } \right)\overrightarrow {q} } \right)\, = \, - \frac{\nabla p}{{\rho_{f} }} + v_{nf} \nabla^{2} \overrightarrow {q} - \frac{{\mu_{nf} }}{{\rho_{nf} K}}\overrightarrow {q} - \frac{{\sigma_{nf} }}{{\rho_{nf} }}\overrightarrow {q} .$$

The modified system of equations for mass conservation and the sustained linear momentum^34–36^ are stated as

3 $$\frac{\partial u}{{\partial x}} + \frac{\partial v}{{\partial y}}\, = \,0,$$

4 $$u\frac{\partial u}{{\partial x}} + v\frac{\partial u}{{\partial y}}\, = \, - \frac{1}{{\rho_{f} }}\frac{\partial p}{{\partial x}} + \frac{{\mu_{nf} }}{{\rho_{nf} }}\frac{{\partial^{2} u}}{{\partial y^{2} }} - \frac{{\mu_{nf} }}{{\rho_{nf} K}}u - \frac{{\sigma_{nf} B_{0}^{2} }}{{\rho_{nf} }}u\, = \,0,$$

and energy equation is

5 $$u\frac{\partial T}{{\partial x}} + v\frac{\partial T}{{\partial y}}\, = \,\chi \frac{{\partial^{2} T}}{{\partial y^{2} }} - \frac{1}{{\left( {\rho C_{p} } \right)_{nf} }}\frac{{\partial q_{r} }}{\partial y},$$

here (*u*, *v*) determines the velocities in the *x* and *y* axis correspondingly, dynamic viscosity of nanofluid is represented as $\mu_{nf}$, $\rho_{nf}$ is the nanofluids density, permeability of the fluid is denoted as *K*, $\sigma_{nf}$ is the electrical conductivity of the nanofluid, $\chi \, = \,\frac{{\kappa_{nf} }}{{\left( {\rho C_{p} } \right)_{nf} }}$ is the thermal diffusivity of the nanofluid, whereas thermal conductivity of nanofluid is $\kappa_{nf}$, $\left( {\rho C_{p} } \right)_{nf}$ acts as heat capacitance of nanofluids.

The related boundary conditions^3–5^ are

6 $$\left. \begin{gathered} u\left( {x,y} \right)\, = \,d\alpha x + \xi_{1} \frac{\partial u}{{\partial y}} + \xi_{2} \frac{{\partial^{2} u}}{{\partial y^{2} }},\,\quad v\, = \,v_{c} ,\quad u\left( {x,\infty } \right)\, \to 0,\,\,\,\,\,\,\,\,\,\, \hfill \\ \theta \left( 0 \right)\, = \,0,\,\quad \theta \left( 0 \right)\, = \,1 + \xi \theta{\prime} \left( 0 \right). \hfill \\ \end{gathered} \right\},$$

where corresponding slip coefficients of the first and second orders are represented through the quantities $\xi_{1}$ and $\xi_{2}$. $d$ is the stretching/shrinking parameter or proportionate shearing at the border. $\xi$ is the temperature jump constant to be determined later. Moreover $v_{c}$ determines mass transpiration factor, represents the suction or blowing depending on $v_{c} > 0$ or $v_{c} < 0$ respectively.

Implementing the following similarity conversation^37^

7 $$\begin{aligned} & u\, = \,\alpha x\frac{\partial f}{{\partial \eta }},\,\quad \,v\, = \, - \sqrt {\alpha v_{f} } f\left( \eta \right)\,,\quad \eta \, = \,\sqrt {\frac{\alpha }{{v_{f} }}} y, \\ & \theta \left( \eta \right)\, = \,\frac{{T - T_{\infty } }}{{T_{w} - T_{\infty } }}. \\ \end{aligned}$$

here $\alpha \,$ is the constant. To model the movement of thermal radiation, the Rosseland approximation is utilized. In this approximate model, the heat flow is supposed to be proportional to the temperature differential, and it flows from solid surface to the liquid. As a result of this, the thermal radiation flux $q_{r}$ to be expressed^38–42^ as follows

8 $$q_{r} \, = \, - \frac{{4\sigma^{*} }}{{3k^{*} }}\frac{{\partial T^{4} }}{\partial y},$$

here $\sigma^{*}$ stands for the Stefan-Boltzmann constant along with $k^{*}$ stands for mean absorption coefficient. While $T^{4}$ may conceivably visualized as $T^{4} \, \cong \,4T_{\infty }^{3} T - 3T_{\infty }^{4} ,$ and ensure there are suitable low temperature changes within the flowing fluid. With this, Eq. (8) can be written as

9 $$\frac{{\partial q_{r} }}{\partial y}\, = \, - \frac{{16\sigma^{*} T_{\infty }^{3} }}{{3k^{*} }}\frac{{\partial^{2} T}}{{\partial y^{2} }},$$

employing of similarity conversation in Eqs. (4)–(5), one obtain

10 $$\Gamma_{1} \frac{{\partial^{3} f}}{{\partial \eta^{3} }} + \Gamma_{2} \left\{ {f\left( \eta \right)\frac{{\partial^{2} f}}{{\partial \eta^{2} }} - \left( {\frac{\partial f}{{\partial \eta }}} \right)^{2} } \right\} - \left\{ {K_{1} \Gamma_{1} \frac{\partial f}{{\partial \eta }} + M\frac{\partial f}{{\partial \eta }}} \right\}\, = \,0,$$

11 $$\Gamma_{3} \left( {1 + N_{r} } \right)\frac{{\partial^{2} \theta }}{{\partial \eta^{2} }} + \Pr \Gamma_{4} f(\eta )\frac{\partial \theta }{{\partial \eta }}\, = \,0,$$

here

$$\Gamma_{1} \, = \,\frac{{\mu_{nf} }}{{\mu_{f} }},\,\,\,\Gamma_{2} \, = \,\frac{{\rho_{nf} }}{{\rho_{f} }},\,\,\,\Gamma_{3} \, = \,\frac{{\kappa_{nf} }}{{\kappa_{f} }}\,{\text{and}}\,\Gamma_{4} \, = \,\frac{{\left( {\rho C_{p} } \right)_{nf} }}{{\left( {\rho C_{p} } \right)_{f} }}.$$

$K_{1} \, = \,\frac{{\mu_{f} }}{{\rho_{f} \alpha K}}$ is the inverse Darcy number (porosity parameter).

$M\, = \,\frac{{\sigma_{nf} B_{0}^{2} }}{{\rho_{f} \alpha }},$ acts as coefficient of Magnetic field (Hartmann number).

$\Pr \, = \,\frac{{\mu_{f} \left( {C_{p} } \right)_{f} }}{{k_{f} }}$, be the Prandtl number.

In additionally,

$N_{r} = \,\frac{{16\sigma^{*} T_{\infty }^{3} }}{{3k^{*} k_{f} }}$ is the radiation parameter.

With transformed frontier constraints are

12 $$\begin{gathered} f\left( 0 \right)\, = \,V_{c} ,\,\,\,\,\,\left( {\frac{\partial f}{{\partial \eta }}} \right)_{\eta \, = \,0} \, = \,d + \lambda_{1} \left( {\frac{{\partial^{2} f}}{{\partial \eta^{2} }}} \right)_{\eta \, = \,0} + \lambda_{2} \left( {\frac{{\partial^{3} f}}{{\partial \eta^{3} }}} \right)_{\eta \, = \,0} ,\,\theta \left( \eta \right)\, = \,1 + \xi \frac{\partial \theta }{{\partial \eta }}\,,\,\quad {\text{at}}\,\quad \eta \,{ = }\,{0,} \hfill \\ \left( {\frac{\partial f}{{\partial \eta }}} \right)_{\eta \, \to \infty } ,\,\,\,\,\,\,\,\,\,\theta \left( \eta \right) \to 0,\quad {\text{as}}\quad \,\eta \to \infty , \hfill \\ \end{gathered}$$

here $\lambda_{1} \, = \,\xi_{1} \sqrt {\frac{\alpha }{{v_{f} }}} > 0,$ and $\lambda_{2} \, = \,\xi_{2} \frac{\alpha }{{v_{f} }} < 0$ are first and second order slips parameter, and mass transpiration factor is represented as $V_{c\,} \, = \,\frac{{v_{c} }}{{\sqrt {\alpha v_{f} } }}$.

The effective dynamic viscosity of the nanofluid is^43^ stated as

13a $$\mu_{nf} \, = \,\frac{{\mu_{f} }}{{\left( {1 - \phi } \right)^{2.5} }},$$

nanofluid density is described^44^ as

13b $$\rho_{nf} \, = \,\left\{ {\left( {1 - \phi } \right) + \phi \left( {\frac{{\rho_{s} }}{{\rho_{f} }}} \right)} \right\}\rho_{f} ,$$

the thermal conductivity of nanofluid is defined^45^ as

13c $$\kappa_{nf} \, = \,\frac{{\kappa_{s} + 2\kappa_{f} - 2\phi \left( {\kappa_{f} - \kappa_{s} } \right)}}{{\kappa_{s} + 2\kappa_{f} + \phi \left( {\kappa_{f} - \kappa_{s} } \right)}},$$

effective heat capacitance of the nanofluids denoted^46^ as

13d $$\left( {\rho C_{p} } \right)_{nf} \, = \,\left( {1 - \phi } \right)\left( {\rho C_{p} } \right)_{f} + \phi \left( {\rho C_{p} } \right)_{s}$$

here $\phi$ is the volume fraction of the nanofluid.

## Analytical solution for momentum equation

In the present article, we will show a closed form exact solution of Eq. (10) together with the boundary conditions (12), one may assume the solution has a format as

14 $$f\left( \eta \right)\, = \,A + Bexp\,\left( { - \delta \eta } \right),$$

where $\delta > 0,$ is yet to be estimated latterly on, moreover the constants A and B must be calculated with employing Eq. (12).

presently,

15 $$A\, = \,V_{c} + \left( {\frac{d}{{\delta + \lambda_{1} \delta^{2} - \lambda_{2} \delta^{3} }}} \right),\,\,{\text{with}}\,\,\,{\text{B}}\,{ = }\,\left( {\frac{ - d}{{\delta + \lambda_{1} \delta^{2} - \lambda_{2} \delta^{3} }}} \right),$$

In order obtained following algebraic equation by incorporating Eq. (13) into Eqs. (10):

15a $$\begin{aligned} & \Gamma_{1} \lambda_{2} \delta^{4} - \left( {\Gamma_{1} \lambda_{1} + \Gamma_{2} V_{c} \lambda_{2} } \right)\delta^{3} + \left( {\Gamma_{2} V_{c} \lambda_{2} - \Gamma_{1} - \Gamma_{1} K_{1} \lambda_{2} - M\lambda_{2} } \right)\delta^{2} + \left( {\Gamma_{2} V_{c} + \Gamma_{1} K_{1} \lambda_{1} + M\lambda_{1} } \right)\delta \\ & \quad + \left( {\Gamma_{2} d + \Gamma_{1} K_{1} + M} \right)\, = \,0, \\ \end{aligned}$$

Equation (14) rewritten as

16 $$\delta^{4} - p\delta^{3} + q\delta^{2} + r\delta + t\, = \,0,$$

where

17 $$\left. \begin{gathered} p\, = \,\frac{{\Gamma_{1} \lambda_{1} + \Gamma_{2} V_{c} \lambda_{2} }}{{\Gamma_{1} \lambda_{2} }},\,\,\, \hfill \\ \,q\, = \,\frac{{\Gamma_{2} V_{c} \lambda_{2} - \Gamma_{1} - \Gamma_{1} K_{1} \lambda_{2} - M\lambda_{2} }}{{\Gamma_{1} \lambda_{2} }},\,\,\,\,\,\,\,\,\,\,\,\,\,\,\,\,\,\,\,\,\,\, \hfill \\ r\, = \,\frac{{\Gamma_{2} V_{c} + \Gamma_{1} K_{1} \lambda_{1} + M\lambda_{1} }}{{\Gamma_{1} \lambda_{2} }}, \hfill \\ t\, = \,\frac{{\Gamma_{2} d + \Gamma_{1} K_{1} + M}}{{\Gamma_{1} \lambda_{2} }}.\,\,\,\,\,\, \hfill \\ \end{gathered} \right\},$$

Equation (15) gives four real roots which helps to provide solution for the current problem. Within this part we have to obtained an analytic approach to the momentum equation. Next move on to the analytical solution for the temperature equation which is discussed in further section.

## Solution for the temperature region of fluid

Now introduce Eq. (13) in Eq. (11) we obtain as follow

18 $$\Gamma_{3} \left( {1 + N_{r} } \right)\frac{{\partial^{2} \theta }}{{\partial \eta^{2} }} + \Pr \Gamma_{4} \left\{ {A + B\exp \left( { - \delta \eta } \right)} \right\}\frac{\partial \theta }{{\partial \eta }}\, = \,0,$$

In the following section, we use the Laplace transformation to examine the temperature solution in the context of the generalized incomplete gamma function and by framing a novel variable as

$$t\, = \,e^{ - \delta \eta } ,$$ we achieve

19 $$\left( {\Gamma_{3} + N_{r} } \right)t\frac{{\partial^{2} \theta }}{{\partial t^{2} }} + \left( {m - nt} \right)\frac{\partial \theta }{{\partial t}}\, = \,0,$$

where as

20 $$m\, = \,\left( {\Gamma_{3} + N_{r} } \right) - \frac{{\Pr \Gamma_{4} A}}{\delta },\quad n\, = \,\frac{{\Pr \Gamma_{4} B}}{\delta }.$$

with transformed boundary constraints are

21 $$\theta \left( 0 \right)\, = \,0,\,\,\,\,\,\,\,\,\,\,\theta \left( 1 \right)\, = \,1 + \xi \left( {\frac{\partial \theta }{{\partial \eta }}} \right)_{\eta \to 1} .$$

Currently we interpolate Laplace transformation upon respective sides of the Eq. (18), once we achieve

22 $$s\left\{ {n - \left( {\Gamma_{3} + N_{r} } \right)s} \right\}\frac{\partial \Phi }{{\partial s}} + \left\{ {n + \left( {m - 2\left( {1 + N_{r} } \right)s} \right)} \right\}\Phi \left( s \right)\, = \,0,$$

here $\Phi \left( s \right)$ is the Laplace transformation of $\theta \left( t \right)\,$, that is $L\left\{ {\theta \left( t \right)} \right\}\, = \,\Phi \left( s \right)$. To execute final expression, it should be observed that the initial modified boundary condition $\theta \left( 0 \right)\, = \,0$ was used. So as to integrating the Eq. (20), we gathered

23 $$\Phi \left( s \right)\, = \,\frac{c}{{s\left( {s - \frac{n}{{A_{3} + N_{r} }}} \right)^{{1 - \frac{m}{{A_{3} + N_{r} }}}} }}.$$

In which $c$ is the integration constant which is to be determined later. For the sake of introduced the reverse Laplace transformation $L^{ - 1}$ to Eq. (21). Thus, $\frac{m}{{A_{3} + N_{r} }}$ must be confined to be $\frac{m}{{A_{3} + N_{r} }} < 1$. As a result, we have follows

24 $$\theta \left( t \right)\, = \,cL^{ - 1} \left\{ \frac{1}{s} \right\}*L^{ - 1} \left\{ {\frac{1}{{\left( {s - \frac{n}{{1 + N_{r} }}} \right)^{{1 - \frac{m}{{A_{3} + N_{r} }}}} }}} \right\},$$

24a $$\theta \left( t \right)\, = \,\frac{c}{{\Gamma \left( {1 - \frac{m}{{A_{3} + N_{r} }}} \right)}}\left( {1*e^{{\frac{n}{{A_{3} + N_{r} }}t}} t^{{\frac{ - m}{{A_{3} + N_{r} }}}} } \right),$$

while $*$ signifies the convolution property as stated by

25 $$L^{ - 1} \left\{ {\Psi \left( s \right),\,\Theta \left( s \right)} \right\}\, = \,\psi \left( t \right)*\varphi \left( t \right)\, = \,\int\limits_{0}^{t} {\psi \left( \mu \right)\varphi \left( {t - \mu } \right)d\mu ,}$$

In order that $L^{ - 1} \left\{ {\Psi \left( s \right)} \right\}\, = \,\psi \left( t \right)$ with $L^{ - 1} \left\{ {\Theta \left( s \right)} \right\}\, = \,\varphi \left( t \right)$. Consequently Eq. (22) develops as

26 $$\theta \left( t \right)\, = \,\frac{c}{{\Gamma \left( {1 - \frac{m}{{A_{3} + N_{r} }}} \right)}}\int\limits_{0}^{t} {\mu^{{\frac{ - m}{{A_{3} + N_{r} }}}} e^{{\frac{n\mu }{{A_{3} + N_{r} }}}} } d\mu ,$$

Put $\sigma \, = \, - n\mu$ in Eq. (24), we obtain

27 $$\theta \left( t \right)\, = \,\frac{c}{{\Gamma \left( {1 - \frac{m}{{A_{3} + N_{r} }}} \right)}}\left( {\frac{ - 1}{n}} \right)^{{1 - \frac{m}{{A_{3} + N_{r} }}}} \Gamma \left( {1 - \frac{m}{{A_{3} + N_{r} }},0,\frac{ - n}{{A_{3} + N_{r} }}t} \right),$$

now accomplish second modified boundary constraints i.e. $\theta \left( 1 \right)\, = \,1 + \xi \left( {\frac{\partial \theta }{{\partial \eta }}} \right)_{\eta \to 1}$, we acquire the value of $c$ as

28 $$c\, = \,\frac{{\Gamma \left( {1 - \frac{m}{{A_{3} + N_{r} }}} \right)}}{{\left( {\frac{ - 1}{n}} \right)^{{1 - \frac{m}{{A_{3} + N_{r} }}}} \Gamma \left( {1 - \frac{m}{{A_{3} + N_{r} }},\,0,\,\frac{ - n}{{A_{3} + N_{r} }}} \right) - \xi e^{{\frac{n}{{A_{3} + N_{r} }}}} }},$$

currently substitute Eq. (26) into Eq. (25), hence, $\theta \left( t \right)$ is presented in the precise form shown below

29 $$\theta \left( t \right)\, = \,\frac{{\Gamma \left( {1 - \frac{m}{{A_{3} + N_{r} }},\,0,\, - \frac{n}{{A_{3} + N_{r} }}e^{ - \beta \eta } } \right)}}{{\Gamma \left( {1 - \frac{m}{{A_{3} + N_{r} }},\,0, - \frac{n}{{A_{3} + N_{r} }}\,} \right) - \xi \left( { - n} \right)^{{1 - \frac{m}{{A_{3} + N_{r} }}}} e^{{\frac{n}{{A_{3} + N_{r} }}}} }},$$

in this section we obtained exact analytic solution for energy equation in terms of generalized gamma functions with employing Laplace transformation formula. Proceed to following section, where we must discuss the current problem outcomes.

## Interpretation of results

We go into greater detail about the physical aspects of the interest constraints related to the current blood flow models. Setting the numerical value of the Prandtl number $$\Pr$$ is 21 for blood. The range of the parameter taken as $$0.5 \le M \le 5,$$$$0.1 \le \phi \le 0.6,$$$$- 1 \le V_{c} \le 4$$, $$0.1 \le K_{1} \le 5,$$$$0.2 \le \lambda_{1} \le 6,$$$$- 6 \le \lambda_{2} \le 0.5,$$ $$0.1 \le \xi \le 5,$$ and $$0.01 \le N_{r} \le 5$$. The results found to be in good agreement. To analyze the flow and heat transfer characteristics of the nanofluid. The computed analytical results of the velocity, temperature as well as shear stress, rates of heat transfer for a benefits of the regulating circumstances, particularly magnetic field $$\left( M \right)$$, stretching/shrinking strength factor $$\left( d \right)$$, first $$\left( {\lambda_{1} } \right)$$ together with second order $$\left( {\lambda_{2} } \right)$$ Navier slip conditions, mass transpiration parameter $$\left( {V_{c} } \right)$$, inverse Darcy number parameter $$\left( {K_{1} } \right)$$, Prandtl number $$\left( {\Pr } \right)$$ together with radiation parameter $$\left( {N_{r} } \right)$$ are demonstrated graphically. Also the physical properties of nanofluids in base fluid are mentioned in the Table 1. Validation of present study and already existing works as shown in Table 2.

**Table 1:** Thermo-physical properties of nanofluid^35,47–50^.

Sl. No.	Nanofluid/base fluid	$\rho$ $\left( {{\text{kg}}/{\text{m}}^{3} } \right)$	$\kappa$ $\left( {{\text{W}}/{\text{mK}}} \right)$	$C_{p}$ $\left( {{\text{J}}/{\text{kg}}\,{\text{K}}} \right)$
1.	Blood	1053	0.492	3594
2.	Silver	10,500	429	235
3.	Copper	8933	401	385

**Table 2:** Comparision of the current research and relevant studies by other authors.

Related works by other authors	Fluids	Value of $\delta$
Mahabaleshwar^34^	Non-Newtonian	$f\left( \eta \right) = A_{1} + B_{1} \exp \left( { - \delta \eta } \right)$ Where $A_{1} = V_{c} + \frac{d}{{\delta + \Gamma_{1} \delta^{2} - \Gamma_{2} \delta^{3} }},$ $B_{1} = - \frac{d}{{\delta + \Gamma_{1} \delta^{2} - \Gamma_{2} \delta^{3} }}.$
Fang^51^	Non-Newtonian	$f\left( \eta \right) = a + b\exp \left( { - \beta \eta } \right)$ Where $a = s + \frac{1}{{\beta + \Gamma \beta^{2} - \delta \beta^{3} }},$ $b = - \frac{1}{{\beta + \Gamma \beta^{2} - \delta \beta^{3} }}.$ Pr Present Present work Non-NewtonianPp Prese
Present work	Non-Newtonian	$f\left( \eta \right) = A + B\exp \left( { - \delta \eta } \right)$ Where $A = V_{c} + \frac{d}{{\delta + \lambda_{1} \delta^{2} - \lambda_{2} \delta^{3} }}$, $B = - \frac{d}{{\delta + \lambda_{1} \delta^{2} - \lambda_{1} \delta^{3} }}$

**Figure 1:**
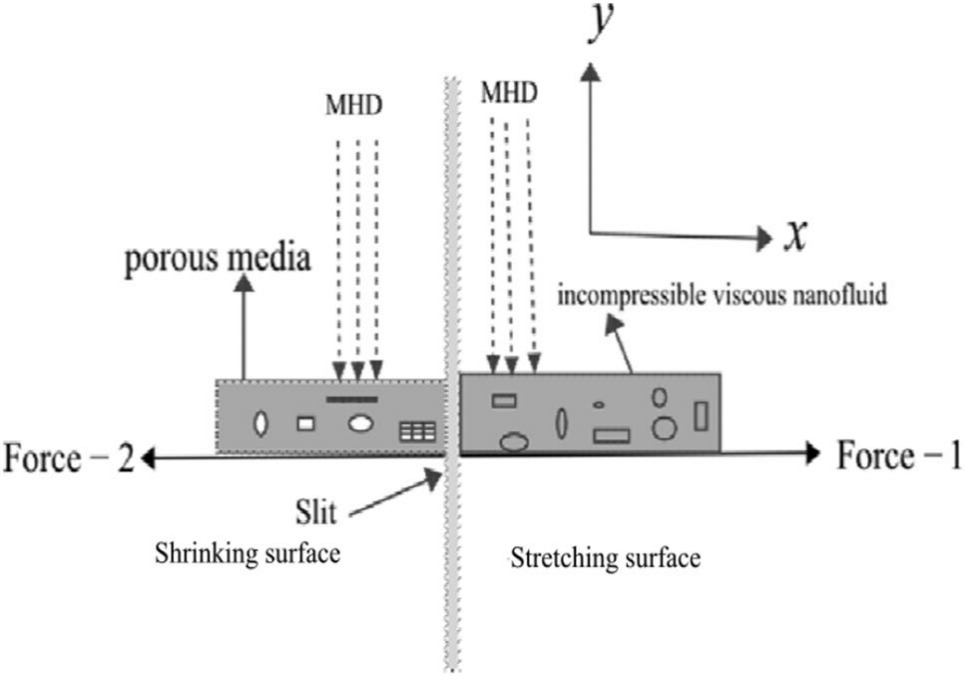
Physical mechanism of the flow problem.

**Figure 2:**
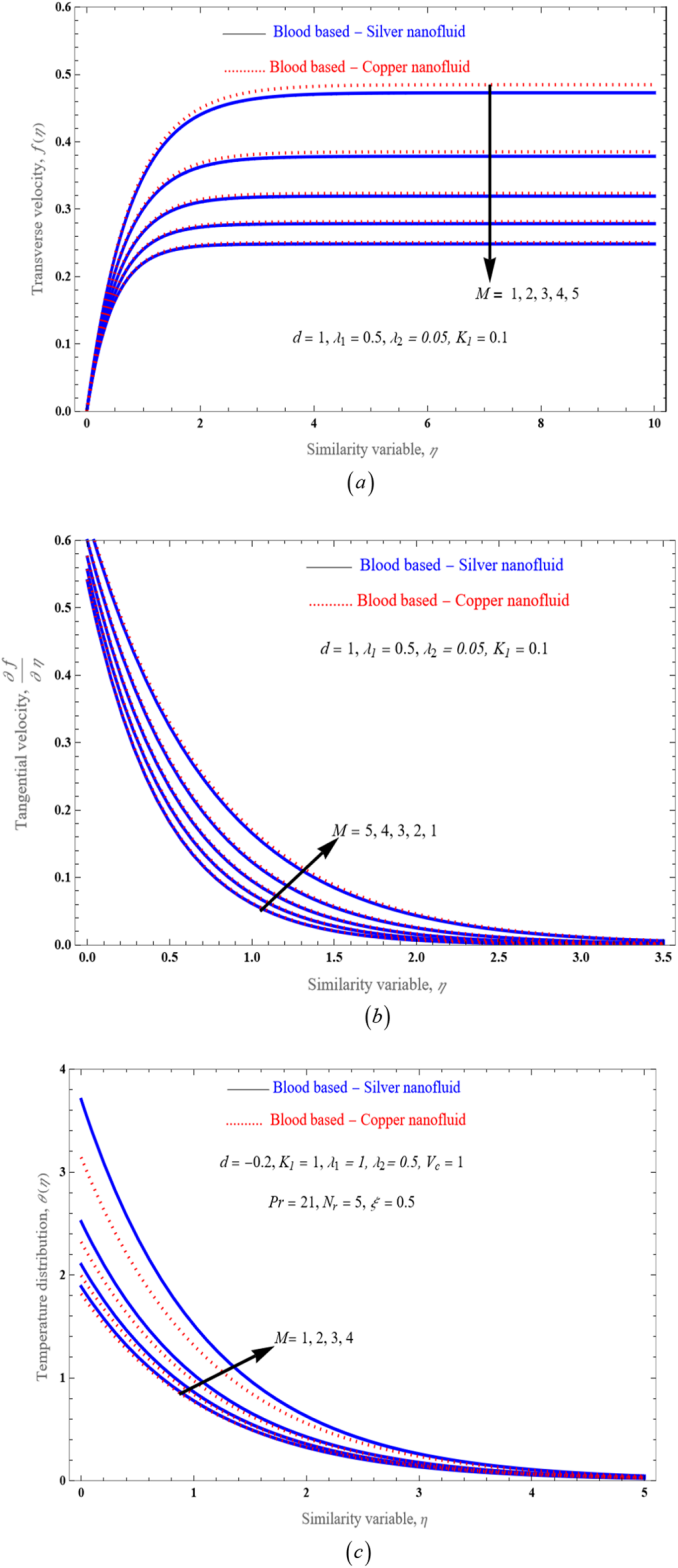
An impact of magnetic field *M* on velocity distribution (**a**,**b**) and temperature distribution (**c**).

The physical mechanism of the problem is given in the Fig. 1. The stimulus of magnetic parameter $M$ on velocity as well as temperature profile is demonstrated through a Fig. 2a–c, which illustrates the role of hartmann number upon velocity outline $f\left( \eta \right),\,\,\,\frac{\partial f}{{\partial \eta }}$, with thermal distribution $\theta \left( \eta \right)$. Figure 2a,b It is observed that rising strength of hartmann number diminishes momentum of the fluid. Hence magnetic field generates force which acts reverse direction. The flow field encounters resistance from this force (Lorentz force) and rate of transport is minimized considerably by keeping the values at $d = 1,\,\lambda_{1} = 0.5,\,\lambda_{2} = 0.05,$ and $K_{1} = 0.1$. The mobility of the nanoparticles inside the surface is reduced by increasing the intensity of the hartmann number, which also has an adverse effect on the bloods viscosity. Also we noticed that copper-blood nanofluid has more velocity distribution as compared with silver-blood nanofluid. Figure 2c depicts the changes of the nanofluid hartmann number on temperature distribution. It has been noted that as hartmann number broadening, the temperature also growing. Due to nanoparticles, bulk temperature as well as thermal boundary layer develops in the nanoparticles because of its more heat conductance. Besides silver nanoparticles has more temperature than that of copper nanoparticles, in view of the fact that silver has a higher heat conductance than copper nanoparticles.

**Figure 3:**
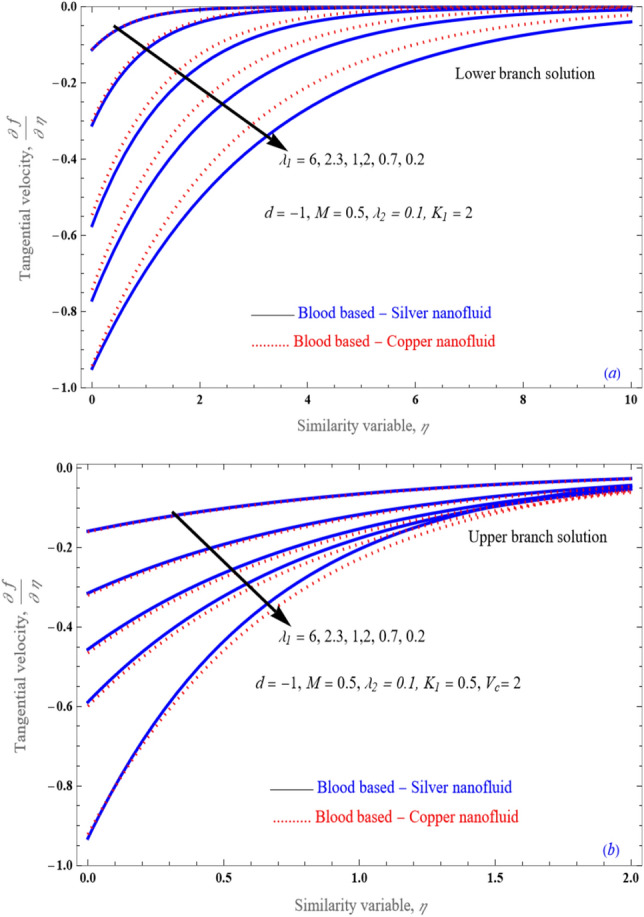
An impact of first order Navier slip $\lambda_{1}$ on (**a**) transverse velocity and (**b**) tangential velocity profile.

Figure 3a,b presents the tangential velocity distribution for several desirability of the primary order slip $\lambda_{1}$ parameter with keeping the values at $d = - 1,\,M = 0.5,\,\lambda_{2} = 0.1,\,K_{1} = 0.5,\,$ and $V_{c} = 2$. This graph apparently express that the lowering the Navier slip, upshots in the diminishes the velocity barrier. Here perceives that width of the nanofluid velocity boundary subject to upper solution branch is reduced as correlated to the lower solution branch. Likewise, one can observe the relationship between the growing velocity boundary width and improvements in the stretching/shrinking factor under stated slip parameter with mass suction parameter. Consequently, decline in mass suction causes a reduction in the acceleration border for other static physical parameters. Although when mass injection is included, the mobility barrier grows as the slip parameter's values rise. As a result, the slip parameter has a big impact on the flow structure and indeed the rate of change in mobility boundary layer width.

**Figure 4:**
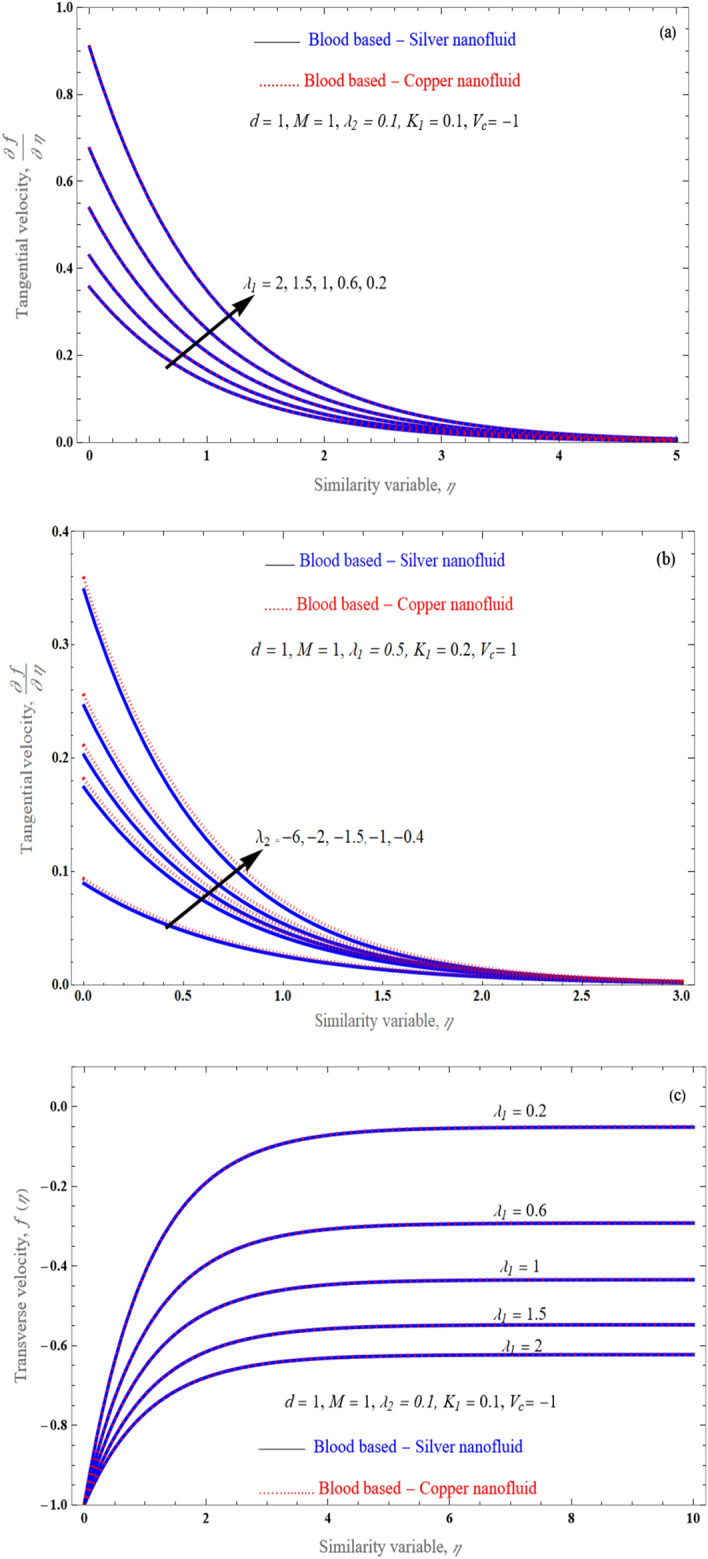
An impact of slip parameter $\lambda_{1}$ and $\lambda_{2}$ on velocity profile for stretching surface.

The impact of the velocity slip parameters $\lambda_{1} \,\,{\text{and}}\,\,\,\lambda_{2}$ on the velocity profile is displayed in the Fig. 4a–c for the silver and copper nanofluids respectively. With rising values of slip parameters, a decline in the dimensionless velocity field is shown in the Fig. 4a–c. when the nanofluids speed lowers and also the velocity slip parameter improves. This is due to truly under slip effects, the movement of the stretched surface is different from the speed of the nanofluid flow nearer to the surface.

**Figure 5:**
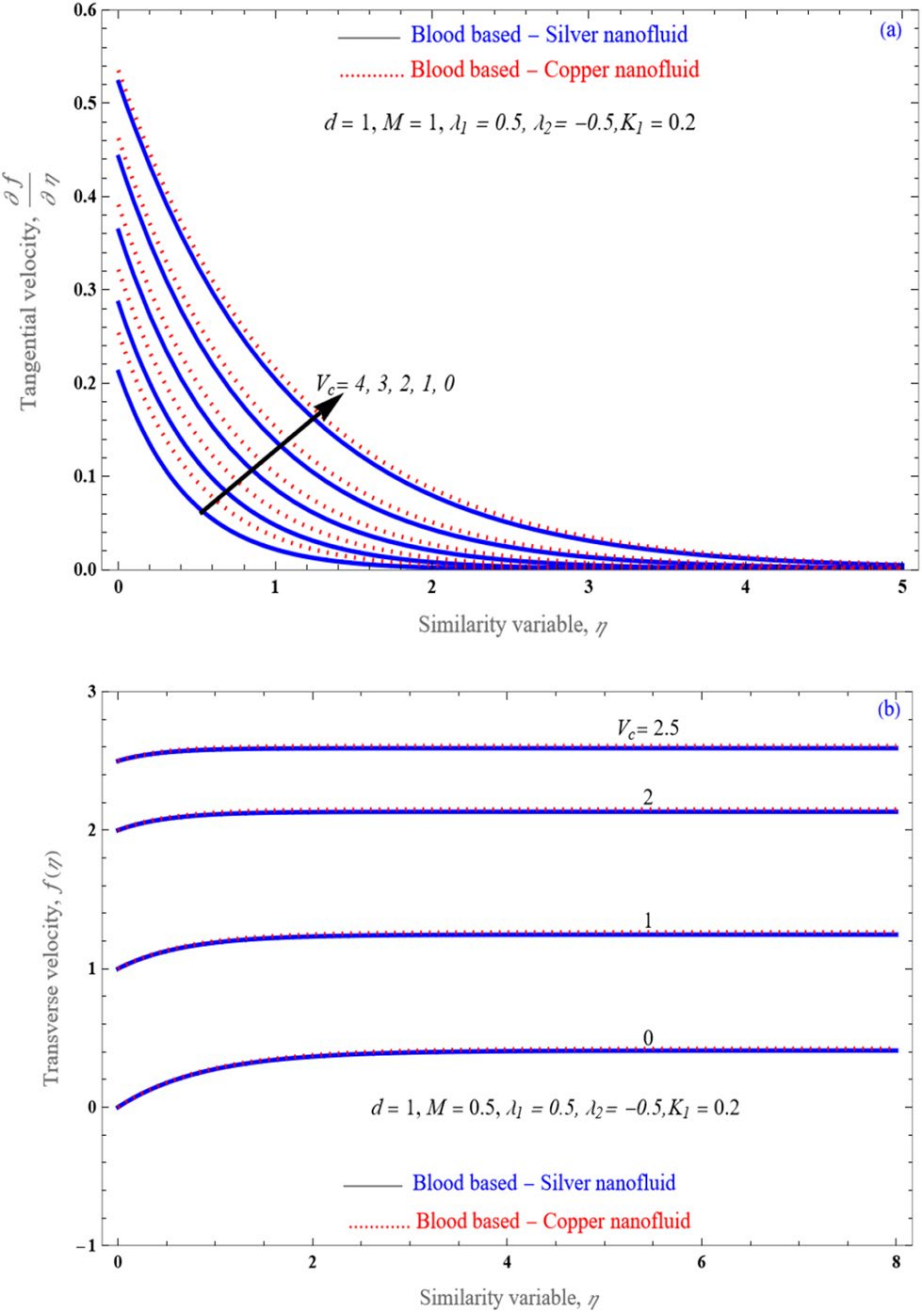
An impact of $V_{c}$ on (**a**) velocity profile and (**b**) tangential velocity profile for stretching case.

Figure 5a be drawn to concluded that influence of mass suction/injection factor $V_{c}$ upon velocity distribution. It is observed that movement of nanofluid flow is decreases (velocity profile reduces) with uplifting value of the suction/injection parameter. Whereas from Fig. 5b it is noticed that acceleration of fluid flow improved as enhancing the quantity of $V_{c}$ value.

**Figure 6:**
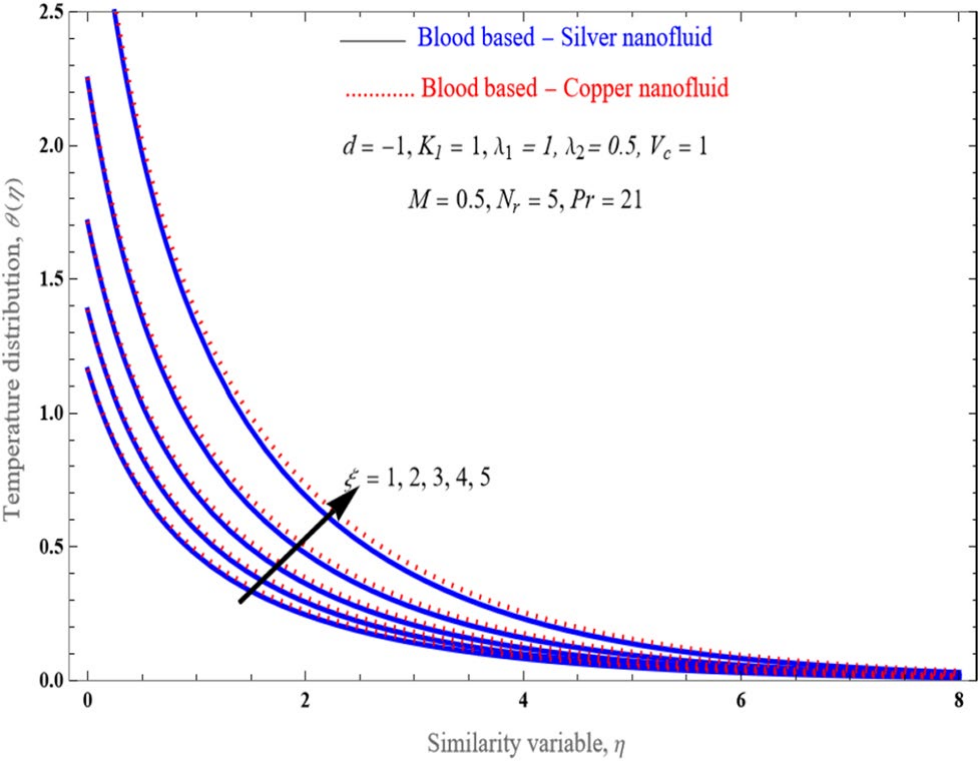
An impact of $\xi$ on temperature profile $\theta \left( \eta \right)$.

Figure 6 illustrates deviations in the temperature field with various values of $\xi$ respectively, while sustaining all other variables at the specified levels. Plot demonstrating that as climbs in the stretching sheet, the temperature velocity does as well. It results in the prediction that the boundary layer will expand as various values improve. It is clearly shows The energy layers increases with an increase in thermal jump.

**Figure 7:**
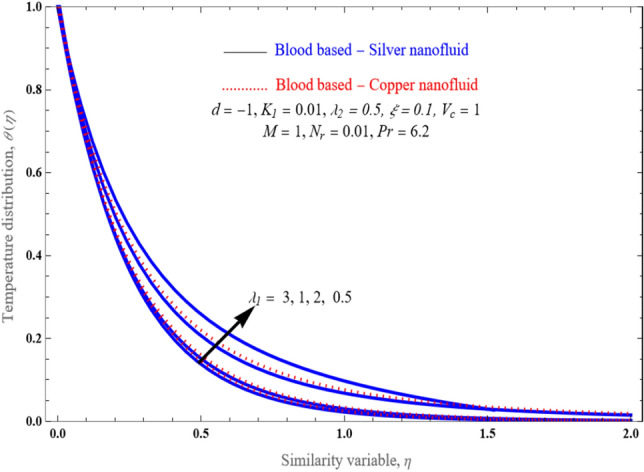
An impact of first order slip factor $\lambda_{1}$ on temperature profile $\theta \left( \eta \right)$.

The slip effect parameter upon temperature is represented in Fig. 7. In the presence of a magnetic field and a thermal jump, the fluid is flowing more freely and consistently as $\lambda_{1}$ raises. Consequently, the profile is more consistent, as well as the heat transmission rate is reduced.

**Figure 8:**
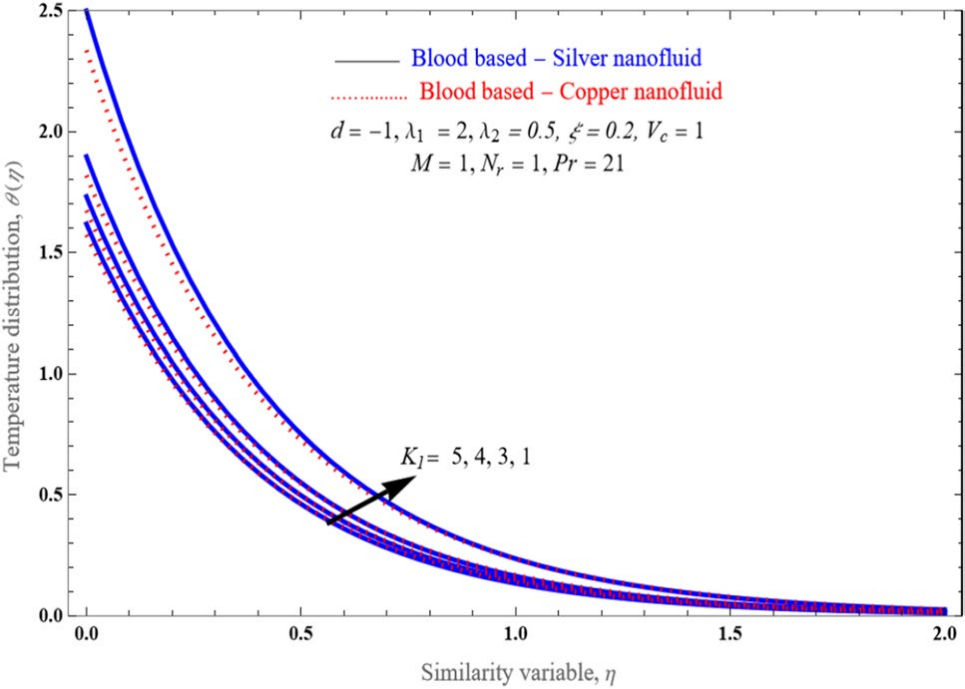
An impact of inverse Darcy number (porosity) on temperature distribution $\theta \left( \eta \right)$.

**Figure 9:**
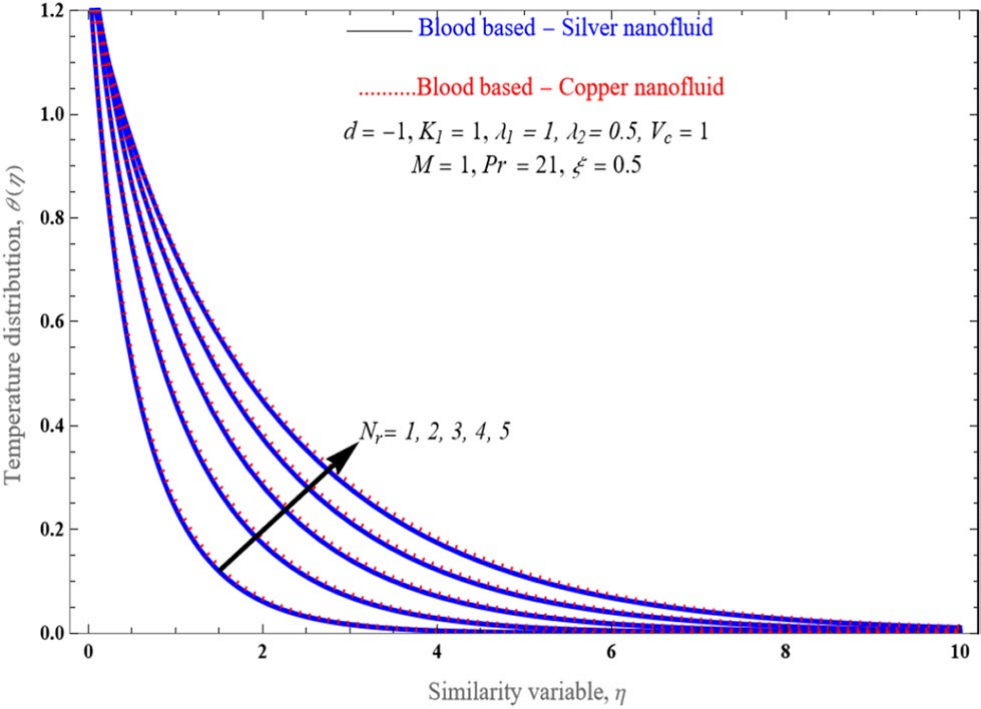
An impact of $N_{r}$ on temperature distribution $\theta \left( \eta \right)$.

Figure 8 illustrates the change in temperature profiles induced by an increment in the value of a porosity component. A boost in the value of the porosity component results in a lowering impact on the temperature distribution. The thickness of the thermal boundary layer becomes thinner with the increase of the porosity parameter. Figure 9 explores how the temperature enhances while thermal radiation accumulates. Although radiation has the consequence of grows the rate at which a fluid's temperature is raised and consequently improving that temperature, the conclusion is subjectively consistent with the assumptions. Since higher radiation parameter values merely raise the surface heat transfer, rendering the fluid hotter.

**Figure 10:**
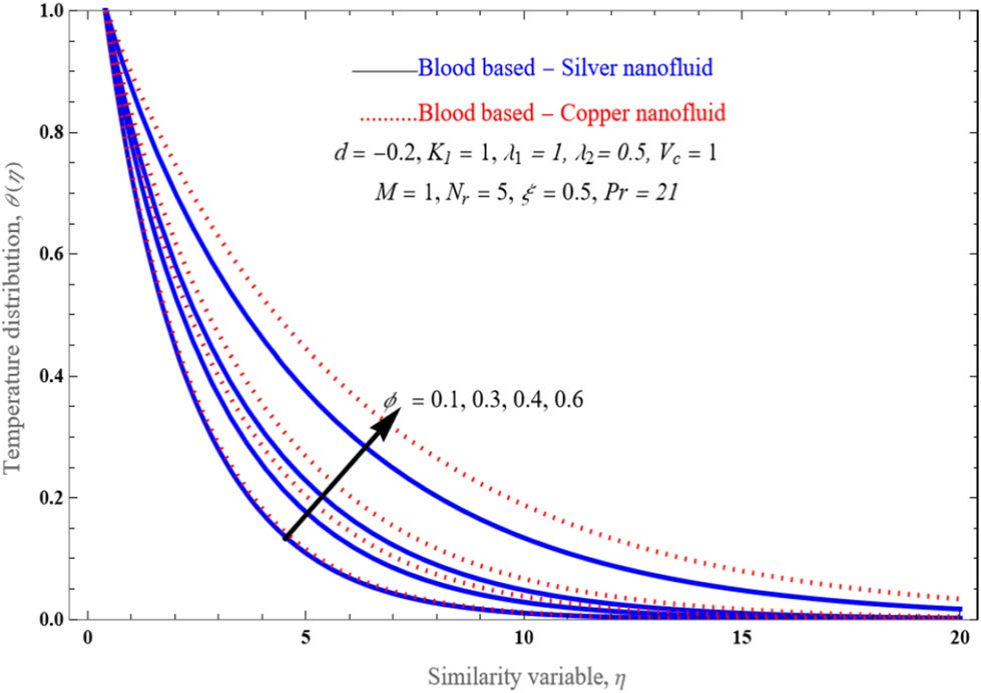
An impact of various values of solid volume fraction $\phi$ on temperature profile.

**Figure 11:**
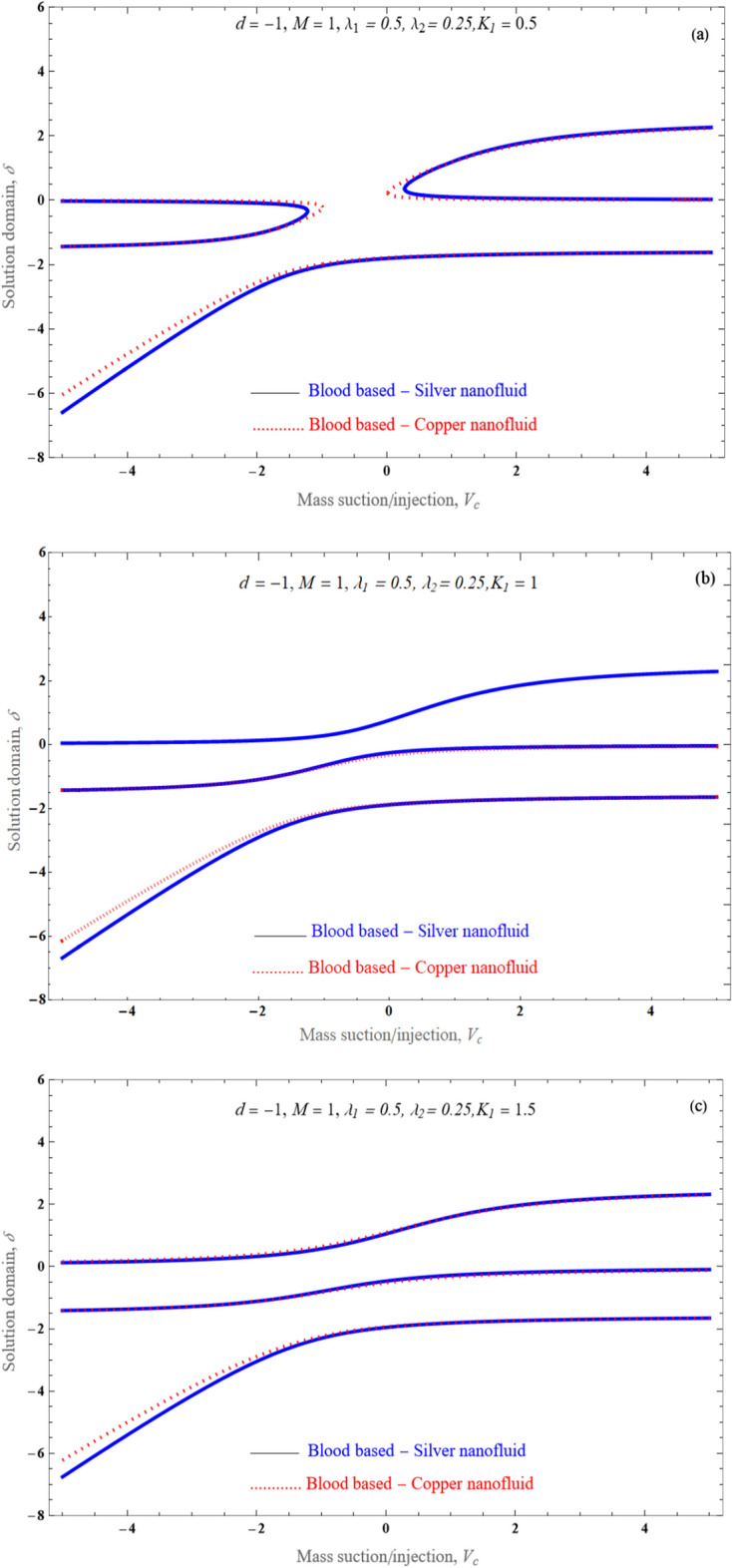
(**a**–**c**) Existence of domain $\delta$ against parameter $V_{c}$ for several values of $K_{1}$.

**Figure 12:**
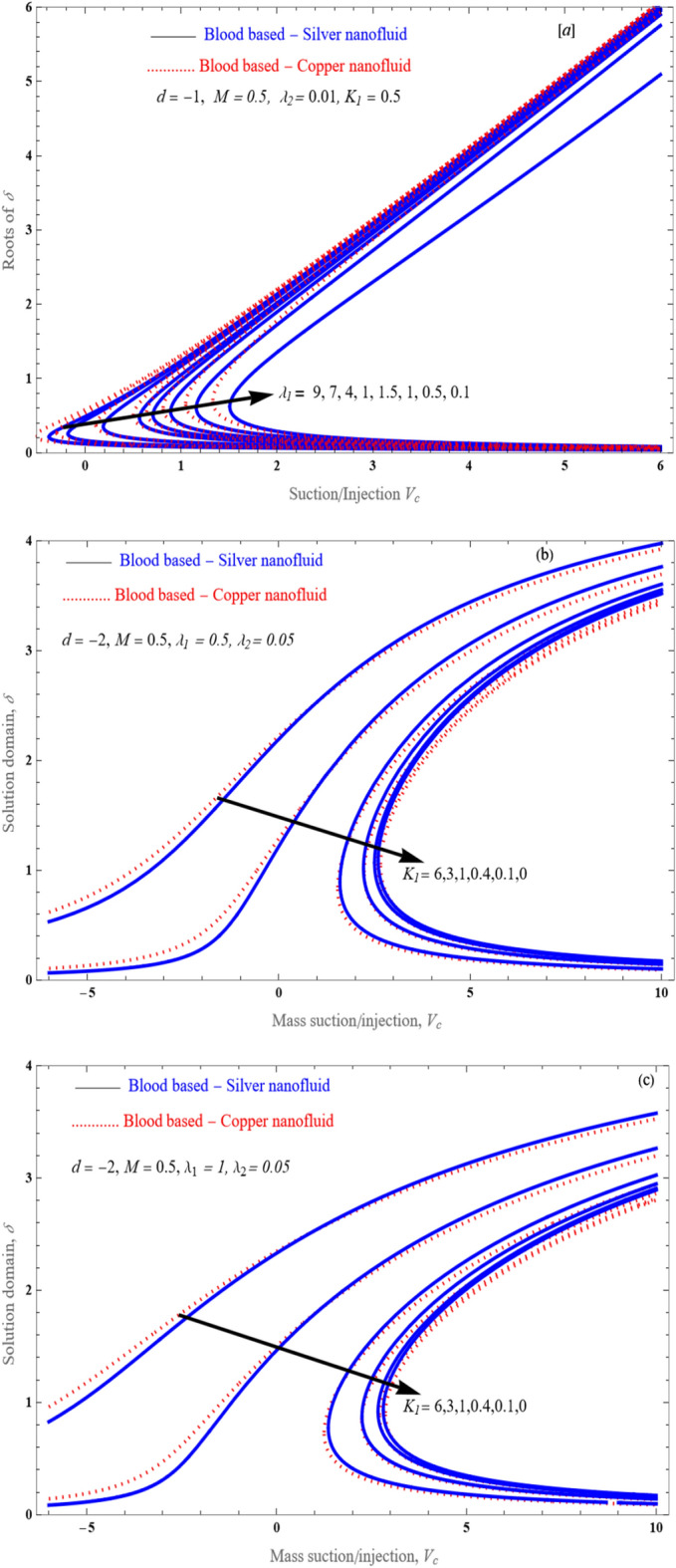
An impact of $\lambda_{1}$(**a**) and $K_{1}$ (**b**,**c**) on solution domain of $\delta$ is plotted against the Mass suction/injection parameter.

An effect of solid volume fraction of the nanofluid on temperature distribution is portrayed in Fig. 10. The nanoparticles volume fraction has improved. since temperature conductance rises among increasing nanoparticle volume values. Physically, this tendency is in line with the premise that copper solid particles have a thermal conductivity that is larger than the silver nanofluids thermal conductivity. The optimal space for $\delta$ against $V_{c}$ as various Porosity $K_{1}$ values is shown in Fig. 11a–c. Expression (15a) is really reduced to a cubic equation by assuming $\lambda_{2} \, = \,0$, and with the right selection of $\lambda_{1}$, and $K_{1}$. From Fig. 11a–c, we observed that the flow solution domain is varied because of nature of the porosity value. An effects $\lambda_{1}$ besides porosity parameter $K_{1}$ upon solution space $\delta$ relative mass suction/injection $V_{c}$ is displayed in Fig. 12a–c. The dispersion curve is forced towards the slit by the appearance of a higher slip. The movement of a viscous liquid in a porous material with slip at a stretched border is very distinct beyond that of a shrinking border.

**Figure 13:**
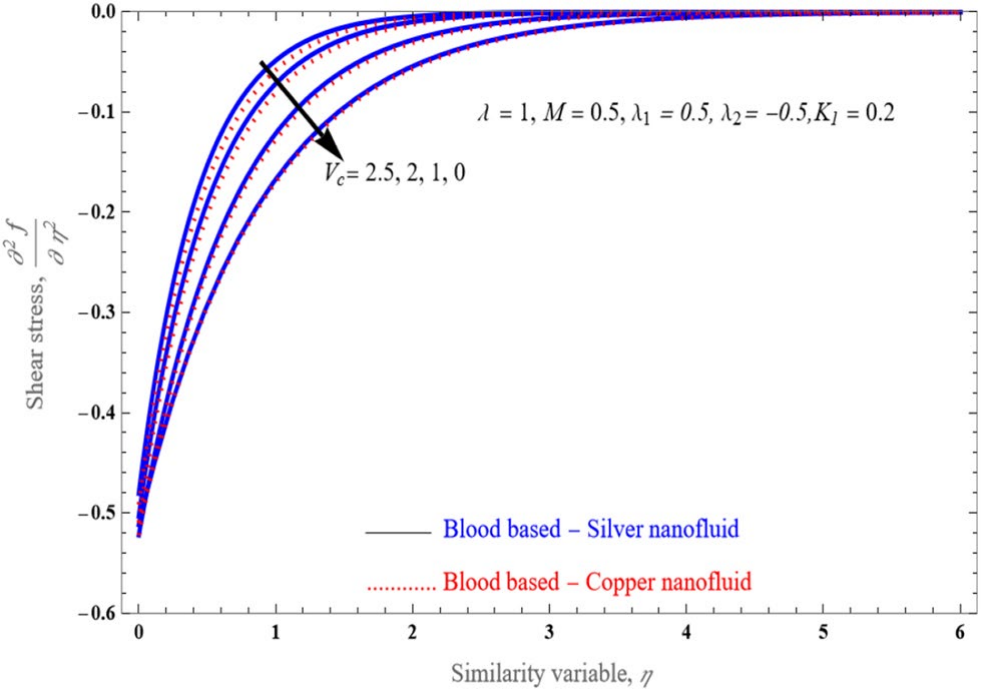
An impact of various values of $V_{c}$ on the shear stress profile for stretched condition and other parameter are fixed.

Figure 13 demonstrated the upshots of several physical characteristics on the shear stress spectrum. In such graph, the overall impacts on the porous solid can be seen at overlap locations for the shear stress spectra. At the wall's boundary, shear increases as the values of $\lambda_{1} ,\,\lambda_{2}$ and porosity parameter rise.

**Figure 14:**
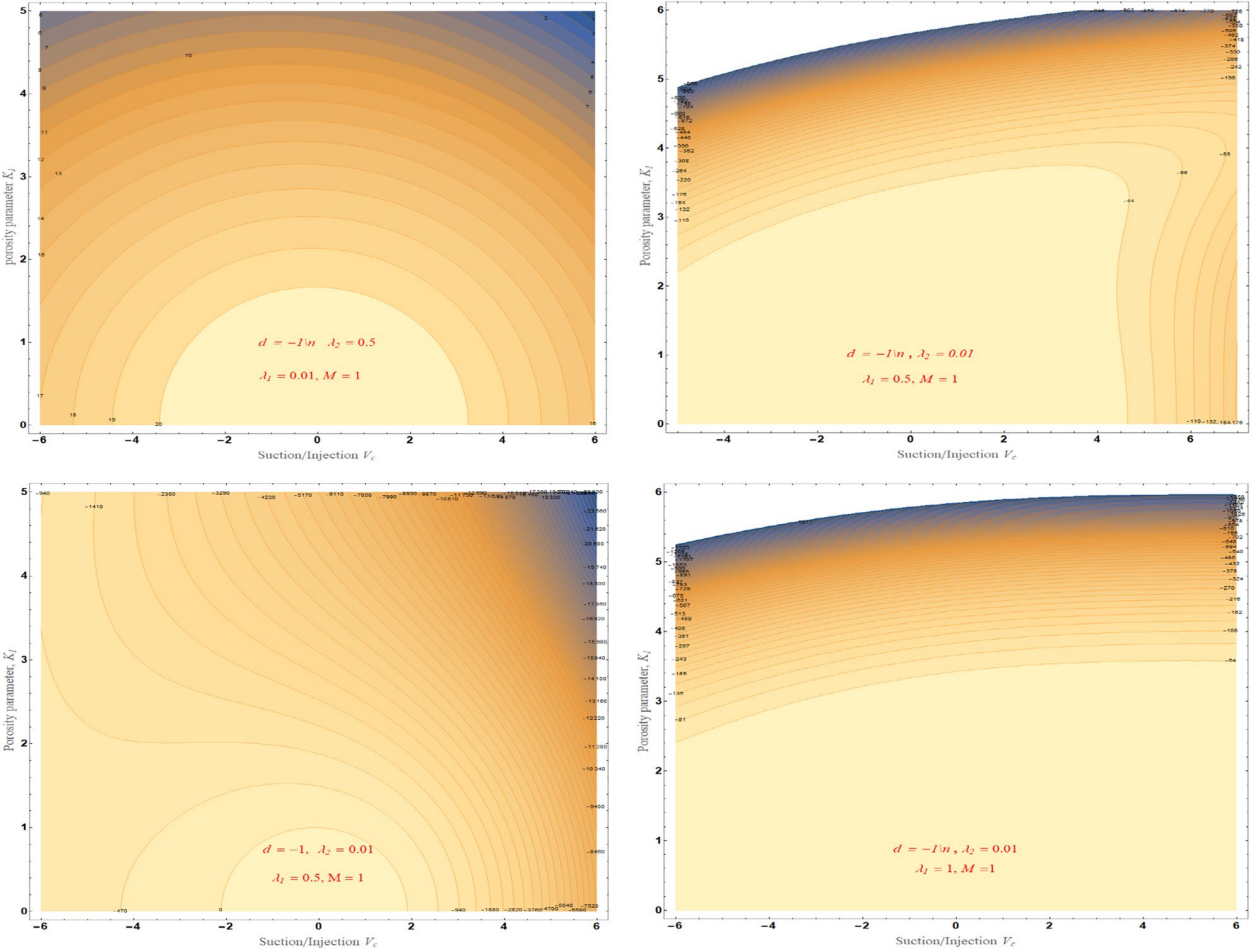
The solution region of porosity parameter $K_{1}$ against mass suction/injection parameter $V_{c}$ for stretched case.

Figure 14a–d demonstrate performance of the solution space $\delta$ of against mass suction/injection parameter $V_{c}$ for varying values of $\lambda_{1}$ with fixed the value at $M = 1$, $\lambda_{2} = 0.01$ and $0.5$. This figures shows the flow patterns of the fluid in the specified region. In fact, by choosing $\lambda_{2} = 0,$ and $M = 0$, Eq. (15a) reduces to a cubic equation and with the proper choice of $\lambda_{1}$, $\Gamma_{1} ,\,\Gamma_{2} ,\,$ and $K_{1}$, and the results are reduced to those obtained by^28,51,52^.

## Concluding remarks

In present study we conduct the theoretical study on laminar stream of viscous incompressible liquid through a penetrable stretching/shrinking sheet under the influence of thermal radiation as well as magnetohydrodynamic effects. Also carry through Navier slip conditions along with mass suction/injection parameter is considered. A similarity conversion is employed to convert the controlling PDEs for momentum and temperature to ODEs. These equations are solved analytically.$Ag$-blood and *Cu*-blood nanofluids are taken into consideration in the study. Plots are employed to examining an impacts of various temperature as well as velocity pattern properties. The comprehensive list of the study’s outputs are:

- As the magnetic parameter *M* increases, the velocity profile and the width of the border layer of momentum decline and also noticed that copper-blood nanofluid have more velocity compared to that of silver-blood nanofluid.
- Silver nanoparticles have a higher temperature than copper nanoparticles have been observed.
- With an increment in the velocity slip parameter, the velocity profile reduces while the temperature profile climbs.
- The Prandtl number has the result of diminishing the width of the thermal boundary layer.
- The temperature of the nanoparticles also rises as the radiation parameter R grows.
- The rate of heat is minimized by the velocity slip parameter.

Several preceding studies serve as the current study limiting points:

- $$\begin{gathered} \lim \hfill \\ M \to 0,\,q_{r} \to 0,\, \hfill \\ T \to 0 \hfill \\ \end{gathered}$${our results $\to$ Mahabaleshwar et al.^34^}
- By setting $\lambda_{2} \, = \,0$, Eq. (15a) reduces to results of Fang et al.^51^, Zhang et al.^52^and Yao et al.^53^.

Further extensions of the current work can be implemented incorporating new physical mechanisms, such as Newtonian or non-Newtonian fluid rheology.

## Validation

The research reveals the effect of Navier’s slip and MHD on laminar boundary layer flow and heat transfer for an incompressible non-Newtonian nanofluid over a porous stretching/shrinking sheet with mass transpiration. In the absences of magnetic field $M = 0$, and solid volume fraction $\phi = 0$ leads to the results of Mahabaleshwar et al.^34^. In the absences of magnetic field $M = 0$, $Da^{ - 1} = 0$, and $\phi = 0$ with absences of temperature leads to the results of Fang et al.^51^.

## Data Availability

Data that support the findings of this study are available from the corresponding author upon reasonable request.

## Abbreviations

*A*: Constant (–)
*B*: Constant (–)
$B_{0}$: Magnetic field (Tesla)
*c*: Constant (–)
$$C_{p}$$: Specific heat ($${\text{JK}}^{ - 1} \,{\text{kg}}^{ - 1}$$)
*d*: Stretching/shrinking parameter (–)
*f*: Velocity similarity function (–)
$$K$$: Permeability ($${\text{N}}/{\text{A}}^{2}$$)
$K_{1}$: Inverse Darcy number (–)
$k^{*}$: Mean absorption coefficient (cm^−^^1^)
*M*: Hartmann factor (–)
$$m,n$$: Constants (–)
$$N_{r}$$: Radiation parameter (–)
*Pr*: Prandtl number (–)
$$q$$: Function of $$\left( {u,v} \right)$$ (–)
$q_{t}$: Rate of change of $q$ with respect to time (–)
$q_{r}$: Radiative heat flux (Wm^−^^2^)
*T*: Fluid temperature (*K*)
*T*_*w*_: Temperature of surface (*K*)
$$T_{\infty }$$: Ambient temperature (*K*)
$u,\,v$: X and y axes velocity coefficients (m s^−^^1^)
$V_{c}$: Mass suction/injection factor (–)
$x,y$: Horizontal and vertical axis (–)
$\left( {\rho C_{p} } \right)_{nf}$: Heat capacitance of nanofluid (${\text{JK}}^{ - 1} \,{\text{kg}}^{ - 1}$)
$\left( {\rho C_{p} } \right)_{f}$: Heat capacitance of fluid (${\text{JK}}^{ - 1} \,{\text{kg}}^{ - 1}$)

## Greek symbols

$\lambda_{1}$: First order slip (–)
$\lambda_{2}$: Second order slip (–)
$$\alpha$$: Constant (–)
$$\Gamma_{1} ,\,\Gamma_{2} ,\,\Gamma_{3}$$,$$\Gamma_{4}$$: Coefficients (–)
$\xi$: Temperature jump constant (–)
$$\xi_{1} ,\,\xi_{2}$$: Slip conditions (–)
$\kappa_{nf}$: Thermal conductivity of nanofluid (${\text{Wm}}^{{ - 1}} /{\text{K}}$)
$\mu_{nf}$: Dynamic viscosity of the nanofluid (Ns m^−^^2^)
$\mu_{f}$: Dynamic viscosity of the fluid (Ns m^−^^2^)
$$\eta$$: Similarity variable (–)
$\theta$: Temperature similarity variable (–)
$\nu_{nf}$: Kinematic viscosity of the nanofluid (${\text{m}}^{2} \,{\text{s}}^{{ - 1}}$)
$\nu_{f}$: Kinematic viscosity of the fluid (${\text{m}}^{2} \,{\text{s}}^{{ - 1}}$)
$\rho_{nf}$: Density of the nanofluid (kg m^−^^3^)
$\rho_{f}$: Fluid density (kg m^−^^3^)
$\sigma_{nf}$: Electrical conductivity of nanofluid (S/m)
$\sigma^{*}$: Stefan–Boltzmann constant (–)
$\phi$: Solid volume fraction (–)
$$\chi$$: Thermal diffusivity (m^2^ s^−^^1^)

## Abbreviations

*nf*: Nanofluid (–)
ODEs: Ordinary differential equations (–)
PDEs: Partial differential equations (–)
MHD: Magnetohydrodynamics (–)

## Code availability

Codes that support the findings of this study are available from the corresponding author upon reasonable request.

## References

[1] Choi SUS, Eastman JA (1995). Enhancing thermal conductivity of fluids with nanoparticles. ASME Publ. Fed..

[2] Eastman JA, Choi SUS, Li S, Yu W, Thompson LJ (2001). Anomalously increased effective thermal conductivities of ethylene glycol-based nanofluids containing copper nanoparticles. Appl. Phys. Lett..

[3] Pozhar LA, Kontar EP, Hua MZC (2002). Transport properties of nanosystems: Viscosity of nanofluids confined in slit nanopores. J. Nanosci. Nanotechnol..

[4] Pozhar LA, Gubbins KE (1997). Quasi hydrodynamics of nanofluids mixtures. Phys. Rev. E..

[5] Pozhar LA (2000). Structure and dynamics of nanofluids: Theory and simulations to calculate viscosity. Phys. Rev. E..

[6] Rashidi MM, Ghahremanian S, Toghraie D, Roy P (2020). Effect of solid surface structure on the condensation flow of argon in rough nanochannels with different roughness geometries using molecular dynamics simulation. Int. Commun. Heat Mass Transf..

[7] Mansoury D, Doshmanziari FI, Rezaie S, Rashidi MM (2019). Effect of Al_2_O_3_/water nanofluid on performance of parallel flow heat exchangers. J. Therm. Anal. Calorim..

[8] Rashad AM, Rashidi MM, Lorenzini G, Ahmed SE, Aly AM (2017). magnetic field and internal heat generation effect on the free convection in a rectangular cavity filled with a porous medium saturated with *Cu*-water nanofluids. Int. J. Heat Mass Transf..

[9] Hatami M, Zhou J, Geng J, Song D, Jing D (2017). Optimization of a lid-driven T-shaped porous cavity to improve the nanofluids mixed convection heat transfer. J. Mol. Liq..

[10] Sheikholeslami M (2019). New computational approach for exergy and entropy analysis of nanofluids under the impact of Lorentz force through a porous media. Comput. Methods Appl. Mech. Eng..

[11] Hatami M, Jing D (2017). Optimization of wavy direct absorber solar collector (WDASC) using Al_2_O_3_-water nanofluid and RSM analysis. Appl. Therm. Eng..

[12] Tang W, Hatami M, Zhou J, Jing D (2017). Natural convection heat transfers in a nanofluid-filled cavity with double sinusoidal wavy walls of various phase deviations. Int. J. Heat Mass Transf..

[13] Hatami M (2017). Nanoparticles migration around the heated cylinder during the RSM optimization of a wavy-wall enclosure. Adv. Powder Technol..

[14] Alves H (1942). Existence of electromagnetic. Nature.

[15] Mahabaleshwar US, Aly EH, Vishalakshi AB (2022). MHD and thermal radiation flow of graphene casson nanofluid stretching/shrinking sheet. Int. J. Appl. Comput. Math..

[16] Mahabaleshwar US, Vishalakshi AB, Anderson HI (2022). Hybrid nanofluid flow past a stretching/shrinking sheet with thermal radiation and mass transpiration. Chin. J. Phys..

[17] Sneha KN, Mahabaleshwar US, Bennacer R, Ganaoui MEL (2021). Darcy brinkman equation for hybrid dusty nanofluid with heat transfer and mass transpiration. Computation..

[18] Anusha T, Mahabaleshwar US, Sheikhnejad Y (2022). An MHD of nanofluid over a porous stretching/shrinking plate with mass transpiration and Brinkmann ratio. Transp. Porous. Media..

[19] Mahabaleshwar US, Anusha T, Hatami M (2021). The MHD Newtonian hybrid nanofluid flow and mass transfer analysis due to super linear-stretching sheet embedded in porous medium. Sci. Rep..

[20] Sneha, K. N., Mahabaleshwar, U. S., Chan, A., Hatami, M. Investigation of radiation and MHD on non-Newtonian fluid flow over a stretching/shrinking sheet with CNTs and mass transpiration. In *Waves Random Complex Media*. 1–20 (2022).

[21] Chahregh HS, Dinarvand S (2020). TiO_2_-Ag/blood hybrid nanofluid flow through an artery with applications of drug delivery and blood circulation in the respiratory system. Int. J. Numer. Methods Heat Fluid Flow..

[22] Misra JC, Chandra S (2018). Effect of couple stresses on electro kinetic oscillatory flow of blood in the microcirculatory system. J. Mech. Med. Biol..

[23] Ghassemi M, Shahidian A, Ahmadi G, Hamian S (2010). A new effective thermal conductivity model for a bio-nanofluid (blood with nanoparticles Al_2_O_3_). Int. Comm. Heat Mass Transf..

[24] Shankar DS, Lee U (2019). Mathematical modelling of pulsatile flow of non-Newtonian fluid in stenosed arteries. Commun. Nonlinear Sci. Numer. Simul..

[25] Nadeem S, Akbar NS (2011). Influence of heat and chemical reactions on Walter’s B fluid model for blood flow through a tapered artery. J. Taiwan Inst. Chem. Eng..

[26] Shahzadi I, Nadeem S (2017). inclined magnetic field analysis for metallic nanoparticles submerged in blood with convective boundary condition. J. Mol. Liq..

[27] Ijaz S, Nadeem S (2018). Consequence of blood medicated nano transportation as drug agent to attenuate the atherosclerotic lesions with permeability impacts. J. Mol. Liq..

[28] Rehman KU, Malik MY, Zehra I, Alqarni MS (2019). Group theoretical analysis for MHD flow fields: A numerical result. J. Braz. Soc. Mech. Sci. Eng..

[29] Bilal S, Malik MY, Awais M, Rehman KU, Hussain A, Khan I (2018). Numerical investigation on D viscoelastic fluid due to exponentially stretching surface with magnetic effects: An application of non-Fourier flux theory. Neural Comput. Appl..

[30] Rehman KU, Malik MY, Makinde OD, Malik AA (2017). A comparative study of nanofluids flow yields by an inclined cylindrical surface in a double stratified medium. Eur. Phys. J. Plus..

[31] Ali U, Alqahtani AS, Rehman KU, Malik MY (2019). On Cattaneo-Christov heat flux analysis with magnetohydrodynamic and heat generation effects in a Carreau nanofluid over a stretching sheet. Rev. Mex. Fis..

[32] Rehman KU, Alshomrani AA, Malik MY (2018). Carreau fluid flow in a thermally stratified medium with heat generation/absorption effects. Case Stud. Therm. Eng..

[33] Rehman KU, Khan AA, Malik MY, Ali U, Naseer M (2017). Numerical analysis subjected to double stratification and chemically reactive species on williamson dual convection fluid flow yield by an inclined stretching cylindrical surface. Chin. J. Phys..

[34] Mahabaleshwar US, Kumar PNV, Nagaraju KR, Bognár G, Nayakar SNR (2019). A new exact solution for the flow of a fluid through porous media for a variety of boundary conditions. Fluids..

[35] Rajagopal KR (2007). On a hierarchy of approximate models for flows of incompressible fluids through porous solids. Math. Model. Methods Appl. Sci..

[36] Mahabaleshwar US, Maranna T, Sofos F (2022). Analytical investigation of an incompressible viscous laminar Casson fluid flow past a stretching/shrinking sheet. Sci. Rep..

[37] Eldabe N, Zeid MA (2013). Thermal diffusion and diffusion thermo effects on the viscous fluid flow with heat and mass transfer through porous medium over a shrinking sheet. J. Appl. Math..

[38] Vishalakshi AB, Maranna T, Mahabaleshwar US, Laroze D (2022). An effect of MHD on non-Newtonian fluid flow over a porous stretching/shrinking sheet. Appl. Sci..

[39] Maranna T, Sneha KN, Mahabaleshwar US, Sarris IE, Karakasidis TE (2022). An effect of radiation and MHD Newtonian fluid over a stretching/shrinking sheet with CNTs and mass transpiration. Appl. Sci..

[40] Zhu J, Liu Y, Cao J (2021). Effect of second-order velocity slip and the different spherical nanoparticles on nanofluid flow. Symmetry..

[41] Maranna T, Mahabaleshwar US, Perez LM, Manca O (2023). Flow of viscoelastic ternary nanofluid over a shrinking porous medium with heat source/sink and radiation. Therm. Sci. Eng. Prog..

[42] Mahabaleshwar US, Maranna T, Perez LM, Ravichandra Nayakar SN (2023). An effect of magnetohydrodynamic and radiation on axisymmetric flow of non-Newtonian fluid past a porous shrinking/stretching surface. J. Magn. Magn. Mater..

[43] Hassain ST, Nadeem S, Ul Haq R (2014). Model based analysis of micropolar nanofluid flow over a stretching surface. Eur. Phys. J. Plus..

[44] Chamkha AJ, Aly AM (2010). MHD free convection flow of a nanofluid past a vertical plate in the presence of heat generation or absorption effects. Chem. Eng. Commun..

[45] Mahdy A (2012). Unsteady mixed convection boundary layer flow and heat transfer of nanofluid due to stretching sheet. Nuclear. Eng. Des..

[46] Benos LT, Polychronopoulos ND, Mahabaleshwar US, Lorenzini G, Sarris IE (2021). Thermal and flow investigation of MHD natural convection in a nanofluid-saturated porous enclosure: an asymptotic analysis. J. Therm. Anal. Calorim..

[47] Mahabaleshwar US, Kumar PNV, Shermet M (2016). Magnetohydrodynamics flow of a nanofluid driven by a stretching/shrinking sheet with suction. Springer Plus..

[48] Siddiqui A, Shankar B (2018). Thermal radiation and slip effects on MHD flow and heat transfer of a Casson nanofluid over a stretching sheet. J. Nanofluids..

[49] Hayat T, Kiran A, Imtiaz M, Alsaedi A (2016). Hydromagnetic mixed convection flow of copper and silver water nanofluid due to curved stretching sheet. Results Phys..

[50] Shahzad F, Jamshed W, Aslam F, Bashir R, Tag EI Din ESM, Khalifa HAE-W, Alanzi AM (2022). MHD pulsatile flow of blood-based silver and gold nanoparticles between two concentric cylinders. Symmetry..

[51] Fang T, Aziz A (2010). Viscous flow with second order slip velocity over a stretching sheet. Zeitschrift Fur Naturforschung A..

[52] Fang TG, Zhang J, Yao SS (2010). Slip magnetohydrodynamic viscous flow over a permeable shrinking sheet. Chin. Phys. Lett..

[53] Fang T, Yao S, Zhang J, Aziz A (2010). Viscous flow over a shrinking sheet with a second order slip flow model. Commun. Nonlinear Sci. Numer. Simul..

